# Representation learning approach for understanding structured documents

**DOI:** 10.1038/s41598-025-33642-y

**Published:** 2025-12-26

**Authors:** Akkshita Trivedi, Sandeep Khanna, Santanu Chaudhury, Gaurav Harit

**Affiliations:** 1 Department of Computer Science and Engineering, Indian Institute of Technology Jodhpur, Jodhpur, India; 2 School of Computing and Data Sciences, FLAME University, Pune, India; 3 Department of Electrical Engineering, Indian Institute of Technology Delhi, New Delhi, India

**Keywords:** Information extraction, Representation learning, Semantic correlation, Document layout, Computational biology and bioinformatics, Mathematics and computing

## Abstract

Current document understanding methods struggle with complex layouts and fail to grasp the deep logical connections between elements like text, figures, and tables. To address this, we introduce the Document Relationship Entity Embedding Learner (D-REEL). This is a novel representation learning framework designed to model intricate semantic relationships within documents. D-REEL works by generating extraction candidates for each article. It then learns dense vector representations (embeddings) for these candidates. By comparing these embeddings, the system accurately assesses semantic correlations between document fields. This allows it to effectively determine if articles are related, regardless of their position on the page. This approach uniquely combines spatial information with domain specific schemas. This enables precise extraction and robust correlation scoring, even across diverse and irregular document layouts. To quantify these connections, we also propose the Semantic Structural Congruence (SSC). This new metric uses location agnostic localization to measure relationships effectively. Experiments on public datasets show significant improvements in correlation accuracy and extraction performance. We achieved an average mAP increment of 2-3% and SSC improvement of almost 10% for the PRIMA dataset.

Most existing document understanding approaches focus on individual elements such as text, images, and tables in isolation or rely only on simple spatial relationships. However, documents are highly interconnected structures where understanding one component often requires reasoning about its relationship to others^1,2^. For instance, extracting text from a figure caption is not sufficient unless the model also understands how the caption relates to the visual content. Similarly, mathematical equations or technical terms often require supporting context from other sections of the document.

Extracting and correlating structured information from documents has long been a significant challenge in both the fields of computer vision and natural language processing. Early work like Chargrid^3^ has introduced 2D layout extraction. This was followed by LayoutLM^4^, LayoutLMv2^5^, and LayoutLMv3^6^, which added spatial and visual details for better results. Vision transformers^7^ and specialized architectures like DocFormer^8^ and StructText^9^ further demonstrated the potential of multimodal pretraining for visually rich documents. Methods like VILA^10^ and PICK^11^ have attempted structured content extraction, while FormNet^12^ and LAMBERT^13^ introduced structure-aware encodings. More recently, GDP (Generic Document Pretraining)^14^ explored generalizable pretraining for broader document types.

Despite recent advances in document processing, most models are optimized for clean, well-formatted inputs like invoices and standard PDFs. Consequently, performance drops significantly when these models are applied to real-world, noisy documents. Examples include digitized archives or low-quality scans. This failure stems from three core issues. First, unreliable OCR occurs due to degraded visual quality. Second, models have difficulty handling non-standard layouts that feature overlapping or misaligned elements. Third, there is an insufficient ability to model crucial semantic relationships. These relationships, such as linking figures to captions or citations to references, are vital for deeper document understanding. However, they remain underrepresented in current architectures.

To address the aforementioned limitations, we propose the D-REEL (Document Relationship Entity Embedding Learner) framework. D-REEL is specifically designed to capture the logical and semantic connections between components in complex documents, such as scientific papers, newspapers, and invoices. Moving beyond reliance solely on spatial features, D-REEL models six distinct categories of entity relationships, linking elements such as title, sections, figures, tables, equations and captions. Furthermore, we introduce the Semantic Structural Congruence (SSC) metric. This novel approach quantifies the semantic relatedness between document components irrespective of their spatial layout or the presence of visual noise. By integrating neighborhood embeddings, spatial encodings, and vision language transformer modules, D-REEL provides robust layout-agnostic reasoning. For the purposes of the experiment, we have also added annotations to existing datasets to define entity relationships. We validate D-REEL on existing challenging datasets, including noisy scans of historical newspapers and multilingual academic records settings where existing state-of-the-art methods typically struggle. D-REEL consistently achieves superior correlation accuracy and robustness while providing interpretable outputs that explicitly highlight these semantic relationships.

The main contributions of our work are the following

1. *D-REEL Framework* We introduce D-REEL (Document Relationship Entity Embedding Learner), a novel representation learning framework. D-REEL make use of positional embeddings to learn deep semantic correlations and logical connections between diverse document entities.
2. *Semantic Structural Congruence (SSC) Metric* We propose the Semantic Structural Congruence (SSC), a new metric designed to quantitatively assess article and component relationships.
3. *Entity Relationship Dataset Enhancement* We enhance existing document datasets by comprehensively labeling them to explicitly establish entity relationships. Furthermore, we conduct extensive experiments on these augmented datasets to validate the efficacy and robustness of the D-REEL framework.

## Related work

The field of document understanding has evolved rapidly, driven by the increasing availability of large-scale document corpora and the development of deep learning methods capable of modeling multimodal information. Traditional approaches^1,2^ to document processing primarily relied on heuristic-based layout detection, optical character recognition (OCR), and rule-based extraction pipelines. While these methods achieved reasonable accuracy in structured scenarios, they often failed to generalize to complex, visually rich documents such as scientific papers, legal filings, and business reports, where textual content, visual layout, and structural hierarchy are tightly interwoven.

*Document layout analysis and segmentation* Layout analysis serves as a foundational task for document understanding, aiming to identify and segment structural components such as paragraphs, figures, tables, equations, and captions^15^. Building upon the limitations of traditional approaches, early deep learning contributions such as Chargrid^3^ proposed a novel grid-based representation of documents, treating text and spatial layout jointly through convolutional neural networks (CNNs). This grid-based paradigm inspired subsequent works like ViBERTgrid^16^ and M2Doc^17^, which refined structural feature learning by integrating layout cues into deep learning pipelines.

The introduction of transformers revolutionized this domain by enabling hierarchical visual modeling beyond CNN limitations. Swin Transformer^18^ introduced a shifted window approach to process images at multiple scales, demonstrating superior performance for layout detection tasks. This multi-scale approach was further enhanced by InternImage^19^, which incorporated deformable convolutions for fine-grained structural recognition. Recent advances such as DLAFormer^20^ and PP-DocLayout^21^ built upon these foundations by integrating multi-scale attention mechanisms with synthetic training samples, addressing robustness challenges under diverse document layouts.

*Pre-trained Models for multimodal document understanding* The evolution from layout-focused approaches to multimodal understanding led to the development of pre-trained models that dramatically improved performance across document understanding benchmarks. The LayoutLM family^4–6^ pioneered this direction by combining text embeddings with two-dimensional layout features, capturing both semantic and spatial information for better visual-textual alignment.

This foundational work evolved through successive iterations: LayoutLMv2^5^ incorporated image embeddings alongside text and layout signals, while LayoutLMv3^6^ introduced unified masking strategies for enhanced pretraining effectiveness. Complementing the LayoutLM series, DocFormer^8^ explored transformer architectures optimized for document-level reasoning, while LamBERT^13^ advanced layout-aware language modeling to capture fine-grained contextual relationships. StructText^9^ adopted multi-modal transformer frameworks with structured encoding strategies, and FormNet^12^ extended structural modeling to accommodate form-like documents with complex field dependencies.

*Information extraction and structural representation* While pre-trained models excelled at understanding individual document components, the challenge of modeling relationships between elements led to the emergence of graph-based approaches. PICK^11^ leveraged graph learning and convolutional networks to capture key information relationships across document layouts, establishing connections between spatially distributed elements. Building on this relationship modeling paradigm, VILA^10^ grouped visual layout elements to improve structured content extraction from complex scientific PDFs. Beyond document-centric applications, graph-based and multiview representation learning methods have demonstrated strong capabilities in modeling complex relational structures across domains. Transformer-powered graph representation learning has been successfully applied to biological networks for interpretable gene identification^22^, while multiview fusion architectures integrating heterogeneous feature spaces have shown effectiveness in protein complex identification through fuzzy clustering^23^. These cross-domain advances highlight the generality of relational reasoning and multiview integration frameworks that similarly benefit document-level structural understanding.

Recent advancements have focused on higher-order document structures, recognizing that documents contain hierarchical relationships beyond pairwise connections. The Multimodal Tree Decoder^24–27^ addressed table of contents extraction by modeling hierarchical dependencies through tree structures. DocSAM^28^ introduced query decomposition and heterogeneous mixed learning for unified document segmentation, while GDP^14^ emphasized generic document pretraining to enhance cross-domain adaptability, providing foundations for transfer learning in document AI.

*Benchmarks for document AI and scientific understanding* The progression from basic layout analysis to complex relationship modeling necessitated comprehensive evaluation frameworks. While early datasets focused on scanned invoices and receipts, the growing sophistication of document understanding methods demanded benchmarks that capture the complexity of real-world documents. DocGenome emerged as a large-scale benchmark specifically designed for training and evaluating multimodal large language models (MLLMs) on scientific documents, emphasizing fine-grained entity recognition and cross-modal reasoning. Complementing this effort, SFDLA^29^ addresses scientific document layout analysis, providing resources to explore complex relationships between text, figures, equations, and metadata within scientific publications.

*Advances in vision-language integration* The convergence of layout analysis, multimodal pretraining, and relationship modeling culminated in sophisticated vision-language integration approaches. Transformer-based architectures underlying models like ViT^7^ combined with language understanding capabilities demonstrated in LayoutLMv3^6^ showcase the potential of cross-modal pretraining. These unified embeddings encode spatial, textual, and visual cues simultaneously, while robust object detection frameworks like YOLOv5^30^ provide essential backbones for layout element identification, completing the pipeline from detection to semantic understanding.

We propose D-REEL, a representation learning framework that transcends traditional spatial constraints by learning dense embeddings that capture semantic relationships between document fields independent of their types or positions.

**Fig. 1:**
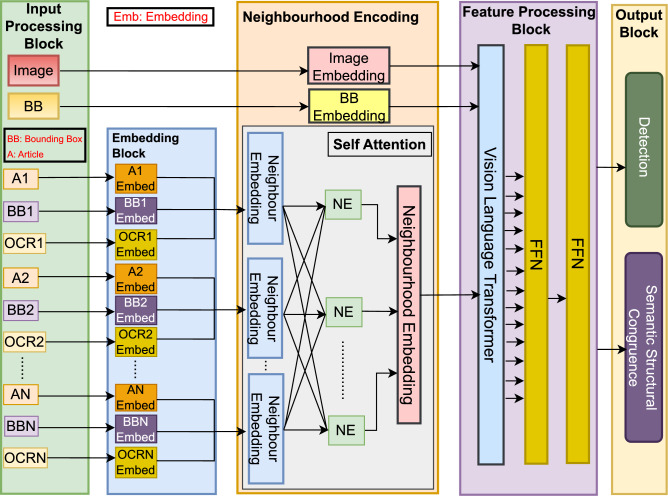
Overview of the proposed DREEL (Document Relationship Entity Embedding Learner) framework. The architecture processes multi-modal inputs (images, bounding boxes, articles, and OCR text) through a dedicated Embedding Block. The Neighbourhood Encoding module utilizes self-attention mechanisms to capture contextual relationships between entities. These features are synthesized in the Feature Processing Block using a Vision Language Transformer and Feed-Forward Networks (FFN) to produce final outputs for detection and semantic structural congruence.

## Document relationship entity embedding learner (D-REEL)

The D-REEL framework illustrated in Fig. 1, designed to effectively integrate spatial and semantic features from document images and textual content, employs a Vision-Language Transformer to model complex correlations across multimodal components. The entire architecture processes a sequence of document elements, transforming an initial feature representation $\textbf{F}_{in}$ through several stages to produce the final task-specific predictions. The framework comprises a structured pipeline: the Input Processing Block, the Embedding Block, the Neighborhood Encoding (NE) Module, the Feature Processing Block, and the final Output Module. The core principle is the continuous transformation of the feature sequence $\textbf{F}$, ensuring tight integration between all architectural components.

### Input processing block & embedding block: generating features

This block acts as the initial stage where different types of data are introduced into the system. The dataset consists of document images, their associated bounding boxes, and articles in the document with corresponding bounding boxes, as well as the OCR text contained within each bounding box. Images provide visual context of articles, bounding boxes define spatial locations within the document, and text segments along with OCR outputs provide the semantic information extracted from these locations. Together, these inputs form a multi-modal dataset that contains both visual and textual information necessary for establishing correlations.

Visual input offers context and setting for text/graphic elements. The spatial structure is critical for defining regions of interest, whereas the textual information provides semantic content critical to understanding meaning. This initial stage generates two sets of features: Local Element Embeddings ($\textbf{F}_{in\_local}$) and Global Context Tokens ($\textbf{E}_{Global}$).

Each article text segment is processed through an embedding model BERT to convert the raw text to a dense vector representation. This vector captures the semantic meaning and contextual nuances of the text. Bounding box spatial features are transformed into embeddings using functions or models trained to consider geometry (area, aspect ratio) and positioning. OCR-extracted text is similarly embedded to integrate the textual content detected from the regions specified by the bounding boxes. Local Element Embeddings ($\textbf{F}_{in\_local}$): For each document element *i* (where *N* is the number of local elements), the features are concatenated:

$$\begin{aligned} E A_i = \text {BERT}(A_i) \end{aligned}$$

$$\begin{aligned} E B B_i = \text {FFN}(\textbf{B B}_i) \end{aligned}$$

$$\begin{aligned} E O C R_i = \text {BERT}(\text {OCR\_text}_i) \end{aligned}$$

The initial local feature vector is $\textbf{F}_{in, i} = [E A_i; E B B_i; E O C R_i]$.The full local sequence is $\textbf{F}_{in\_local} = \{\textbf{F}_{in, 1}, \dots , \textbf{F}_{in, N}\}$.

Global Context Tokens ($\textbf{E}_{Global}$):These features represent the entire document and are held separately before integration in the NE module:

$$\begin{aligned} \textbf{E}_{Img} = \text {ImageEncoder}(\text {Image}) \end{aligned}$$

$$\begin{aligned} \textbf{E}_{DocBB} = \text {FFN}(\text {Document\_BB}) \end{aligned}$$

The global tokens are $\textbf{E}_{Global} = \{\textbf{E}_{Img}, \textbf{E}_{DocBB}\}$.

### Neighborhood encoding (NE) module: contextualized sequence ($\textbf{F}_{NE}$)

The NE module is where all local and global features merge, leveraging positional information and self-attention to create a spatially-aware, fully-contextualized feature sequence. Neighbor Embedding learns spatial dynamics and contextual interactions between bounding boxes and text segments.

#### Neighbor embedding module (local contextualization)

The positional coordinates $p_i$ of the local elements are embedded:

$$\begin{aligned} \text {PosEmbed}(p) = F_{\text {dropout}}(F_{\text {relu}}(F_{\text {linear2}}(F_{\text {dropout}}(F_{\text {relu}}(F_{\text {linear1}}(p)))))) \end{aligned}$$

The base local feature $\textbf{F}_{in, i}$ is enriched with $\text {PosEmbed}(p_i)$ to form the local neighborhood feature $\textbf{F}_{N, i}$:

$$\begin{aligned} \textbf{F}_{N, i} = [\textbf{F}_{in, i} ; \text {PosEmbed}(p_i)] \end{aligned}$$

#### Sequence fusion and self-attention

The neighborhood relationships are modeled using multi-head attention. The Global Context Tokens ($\textbf{E}_{Global}$) are prepended to the sequence of local neighborhood features $\textbf{F}_{N\_local} = \{\textbf{F}_{N, 1}, \dots , \textbf{F}_{N, N}\}$ to form the full feature sequence $\textbf{F}_{N\_full}$:

$$\begin{aligned} \textbf{F}_{N\_full} = [\textbf{E}_{Img}; \textbf{E}_{DocBB}; \textbf{F}_{N, 1}; \textbf{F}_{N, 2}; \dots ; \textbf{F}_{N, N}] \end{aligned}$$

The resulting sequence $\textbf{F}_{N\_full}$ has a length of $N+2$. Multi-Head Attention is then applied to model all relationships (global and local), generating the spatially-aware feature sequence $\textbf{F}_{NE}$:

$$\begin{aligned} \textbf{F}_{NE} = \text {MultiHead}(\textbf{F}_{N\_full}, \textbf{F}_{N\_full}, \textbf{F}_{N\_full}) \end{aligned}$$

### Feature processing block: refined features ($\textbf{F}_{\text {proc}}$)

The feature processing block integrates, Vision Language Transformer which combined visual and textual features, and Feed Forward Network (FFN) consist of two sequential networks that progressively refine the features. This block takes the fully contextualized sequence $\textbf{F}_{NE}$ and subjects it to Vision-Language Transformer layers and sequential FFNs for final feature alignment and refinement.The output of the Transformer layers is denoted $\textbf{F}_{\text {combined}}$:

$$\begin{aligned} \textbf{F}_{\text {combined}} = \text {VLT}(\textbf{F}_{NE}) \end{aligned}$$

This intermediate feature is then passed through two sequential FFNs:

$$\begin{aligned} \textbf{F}_{\text {proc}} = \text {FFN}_2(\text {FFN}_1(\textbf{F}_{\text {combined}})) \end{aligned}$$

The resulting $\textbf{F}_{\text {proc}}$ is the final, task-ready feature sequence of length $N+2$.

### Output block

The Output Block extracts the necessary features from the final sequence $\textbf{F}_{\text {proc}}$ for prediction.

#### Detection component

The Detection component identifies and localizes objects within the input image. The Detection Head predicts bounding boxes for the *N* local elements by using the corresponding part of $\textbf{F}_{\text {proc}}$ (index 3 through $N+2$):

$$\begin{aligned} \hat{\textbf{B}} = \text {DetectionHead}(\textbf{F}_{\text {proc}}[3:N+2]) \end{aligned}$$

$$\begin{aligned} L_{\text {detection}} = \lambda _{loc}L_{loc} + \lambda _{cls}L_{cls} \end{aligned}$$

#### Semantic structural congruence (SSC)

This component quantifies the semantic alignment between visual content and article text. The SSC score, a document-level prediction, is computed using features from $\textbf{F}_{\text {proc}}$. This typically involves using the fused global features (tokens 1 and 2) or a pooled representation $\textbf{F}_{SSC}$ of the entire sequence:

$$\begin{aligned} \textbf{F}_{SSC} = \text {Pooling}(\textbf{F}_{\text {proc}}) \end{aligned}$$

$$\begin{aligned} \text {SSC} = \sigma (f_{\text {corr}}(\textbf{F}_{\text {proc}})) \end{aligned}$$

where the correlation function is:

$$\begin{aligned} f_{\text {corr}}(\textbf{F}_{\text {proc}}) = W^T_{\text {corr}}(\textbf{F}_{\text {vis}}\cdot \textbf{F}_{\text {text}}) + b_{\text {corr}} \end{aligned}$$

The correlation loss is:

$$\begin{aligned} L_{\text {corr}} = -\frac{1}{N}\sum _{i=1}^N[y_i\log (\text {SSC}_i) + (1-y_i)\log (1-\text {SSC}_i)] \end{aligned}$$

#### Combined training objective

The overall objective integrates the two task losses, allowing for shared representation learning across tasks:

$$\begin{aligned} L_{\text {total}} = \alpha L_{\text {detection}} + \gamma L_{\text {corr}} \end{aligned}$$

### Scoring

We formulate the structured data extraction task as a two-stage ranking and optimization problem. Let $\mathcal {D}$ represent a document containing a set of $\mathcal {N}$ candidate entities, denoted as $\mathcal {C} = \{c_1, c_2,...,c_n\}$. Each candidate corresponds to a bounding box and its associated textual and visual features. The objective is to map these candidates to a predefined schema of *M* target fields, $\mathcal {K}= \{k_1, k_2,...,k_n\}$ (e.g., Title, Author, Reference).

*Context-Aware Representation* Visual and textual inputs are first processed through the DREEL framework. Specifically, the Input Processing and Neighbourhood Encoding blocks transform each candidate $c_j$ into a high-dimensional latent representation $h_j \in \mathcal {R}^d$. This embedding $h_j$ is the output of the Vision Language Transformer in the Feature Processing Block, effectively encoding the local visual cues, textual content, and neighborhood structural relationships required for classification.

*Stage I: Independent Candidate Scoring* In the first stage, we define a probabilistic scoring function to evaluate the likelihood of a candidate belonging to a specific field. To ensure that the model focuses on learning robust representations from local context without bias from cross-field dependencies, we model the probability independently for each candidate-field pair. Let $\Phi : \mathcal {R}^d \times \mathcal {K} -> [0,1]$ be a scoring function parameterized by weights $\theta$ realized via the Feed-Forward Networks (FFN) in the Output Block. The confidence score $s_{ij}$ for assigning candidate $c_j$ to field $k_i$ is defined as:

1 $$\begin{aligned} s_{ij} = P(y_{ij} = 1 | h_j, k_i; \theta ) = \sigma (W_i^Th_j + b_i) \end{aligned}$$

where: $y_{ij} \in \{0, 1\}$ is the binary indicator that candidate $c_j$ is the correct extraction for field $k_i$. $W_i$ and $b_i$ are the field-specific weight matrix and bias terms within the FFN. $\sigma (\cdot )$ denotes the sigmoid activation function ensuring the output lies within [0, 1]. This formulation allows the model to decouple feature learning from layout constraints, effectively treating the extraction of each field as an independent binary classification task during the scoring phase.

*Stage II: Assignment and Inference* The second stage functions as an assignment module $\mathcal {A}$ that maps fields to candidates based on the scores computed in Stage I. While the independent scoring provides a confidence map, the final extraction requires a discrete selection.

We employ a greedy maximization strategy to select the optimal candidate for each field. For a target field $k_i$ the predicted candidate $\hat{c}_i$ is determined by maximizing the independent confidence score:

2 $$\begin{aligned} \hat{c}_i = \text {argmax}_{c_j \in \mathcal { C}}(s_{ij}) \end{aligned}$$

## Experimental results and comparison

To evaluate the effectiveness of our proposed D-REEL framework, we conduct extensive experiments across multiple benchmark datasets including DocBank, IIIT-AR-13K, and S2VL. Our experimental setup follows a two-stage approach: first, we pretrain D-REEL on all available datasets to establish robust feature representations, then evaluate performance on document layout detection and corelation tasks. We compare our method against state-of-the-art baselines using standard evaluation metrics including F1-score and mean Average Precision (mAP) and SSC.

### Dataset

We utilize multiple large-scale and diverse document datasets to evaluate both document layout detection and our proposed D-REEL (Document-Relationship Entity Embedding Learner) framework. For D-REEL, we annotate 200 images across datasets using LabelMe^31^ to define entity relationships such as text to image, figure to caption, and header to subsection, enabling deeper semantic modeling beyond traditional layout analysis.

*PubLayNet dataset* The PubLayNet^32^ dataset comprises 360,000 pages with 3.3 million bounding boxes annotated across five categories: text, title, list, table, and figure. It provides a large-scale benchmark for document layout detection and article correlation tasks.

*IIIT-AR-13K dataset* The IIIT-AR-13K^33^ dataset contains 13,000 images covering magazines, flyers, and newspapers with bounding boxes for text, images, logos, and decorative elements, supporting fine-grained document parsing.

*S2-VL dataset* The S2-VL^10^ dataset, part of the S2-VLUE benchmark, offers human-annotated visual layouts for scientific documents across 19 disciplines, averaging 12 text blocks and 90 text lines per page.

*PRImA newspaper dataset * The PRImA^34^ Newspaper Dataset comprises 1,533 historical newspaper images annotated across 15 region classes, with an average of 98–100 regions per page, including 12% overlapping elements and a 70% dominance of text regions.

*German-brazilian newspapers (GBN) dataset* The GBN^35^ dataset contains 152 high-resolution grayscale pages (600 dpi) from eight historical German-language newspapers published in Brazil, annotated with pixel-level labels for text, images, graphics, and separators, posing challenges due to degraded print quality and mixed Fraktur-Latin scripts.

### Experimentation details

We conduct our experiments on an NVIDIA RTX A5000 GPU. The model architecture is based on a transformer encoder with 12 self attention heads, which allows it to effectively capture complex dependencies in the input sequences. The model’s hidden size, intermediate feed forward network size, and other architectural hyperparameters are chosen to strike a balance between model complexity and computational efficiency. To optimize the model, we use the Rectified Adam (RAdam) optimizer with a learning rate of $5 \times 10^{-5}$, which has been shown to improve training stability, especially in transformer based models. The fine tuning process is carried out with a batch size of 30 for all models, and the model is trained for 50 epochs. We employ distributed training across multiple GPUs and utilize mixed precision training to accelerate computation without sacrificing accuracy, making the training process more efficient. These techniques significantly reduce the overall training time while maintaining model performance.

For dataset preparation, all datasets are split into three distinct partitions: the training split (comprising 80% of the dataset) is used to train the model; the validation split (10%) is used to evaluate the model’s performance during training, and the best model is selected based on the lowest hold out loss; and the test split (10%) is used to report the final performance metrics. The evaluation is carried out using standard metrics such as accuracy, precision, recall, and F1-score, computed on the test split to assess the model’s ability to generalize to unseen data.

### Quantitative results

*Precision Result* The empirical results of our comparative analysis demonstrate the superior performance and robustness of our proposed D-REEL model. We evaluated D-REEL against three leading models LayoutLMv3^6^, DocLayout-YOLO^30^, and DocSAM^28^across five diverse public datasets, using Average Precision (AP50) and mean Average Precision (mAP) as our primary metrics. The findings shown in Table 1 confirm that D-REEL outperforms all baseline models across every dataset, establishing a new state-of-the-art in document layout analysis and highlighting the generalized effectiveness of our architecture.

**Table 1:** Detection Metric score across datasets for different document layout models with all embeddings: Image, Layout, OCR.

Dataset / Model	LayoutLMv3^6^		DocLayout-YOLO^30^		DocSAM^28^		LayoutLLM^36^		DocLayLLM^37^		D-REEL	
	AP50	mAP	AP50	mAP	AP50	mAP	AP50	mAP	AP50	mAP	AP50	mAP
PRImA Newspaper Dataset^34^	90.63	89.63	80.12	69.83	84.06	80.69	90.59	90.55	91.05	89.95	92.49	90.64
German-Brazilian Newspapers (GBN) Dataset^35^	90.58	88.93	82.75	70.34	83.12	82.46	90.89	88.80	91.20	89.15	91.72	90.09
S2-VL Dataset^10^	95.76	92.97	95.45	90.12	95.29	90.19	96.50	93.00	96.75	93.60	96.91	95.12
IIIT AR 13K^33^	92.83	93.12	94.39	89.96	84.26	83.89	92.95	92.90	92.97	92.10	93.82	93.28
Publaynet^32^	94.61	93.85	94.98	90.67	95.43	90.73	95.40	93.52	95.55	93.99	95.68	94.07

D-REEL shows particularly strong performance on datasets known for their complex and irregular layouts, such as historical newspapers. On the PRImA^34^ Newspaper Dataset, our model achieves an mAP of 90.64, a significant improvement of nearly a full point over the next-best competitor, LayoutLMv3^6^. This advantage is even more pronounced on the German-Brazilian Newspapers (GBN)^35^ Dataset, where D-REEL’s 90.09 mAP not only surpasses the previous leading model but also shows a substantial leap over other methods, underscoring its enhanced capability in parsing challenging, real-world documents.

This trend of superior performance continues across datasets featuring scientific and contemporary documents. For the S2-VL^10^ and IIIT AR 13K datasets^33^, D-REEL maintains a clear lead, achieving top-tier results of 95.12 mAP and 93.28 mAP, respectively. Notably, on the large-scale Publaynet dataset, D-REEL achieves a remarkable 94.07 mAP, confirming its scalability and effectiveness on extensive and diverse document collections.

*Semantic structural congruence (SSC) score* To evaluate D-REEL core capability of understanding deeper logical connections, we employed the Semantic Structural Congruence (SSC) metric. Table 2 presents a comparative analysis of D-REEL against a comprehensive set of baselines, including recent Large Language Model (LLM) based approaches like LayoutLLM^36^ and DocLayLLM^37^. The findings indicate that D-REEL excels in grasping semantic and structural connections, thereby highlighting its considerable architectural benefits.

**Table 2:** Semantic Structural Congruence (SSC) across datasets for different document layout models using Image, Layout, and OCR embeddings.

Dataset / Model	LayoutLMv3^6^	DocLayout-YOLO^30^	DocSAM^28^	LayoutLLM^36^	DocLayLLM^37^	D-REEL
PRImA Newspaper Dataset^34^	63.59	65.89	65.12	70.95	71.37	81.57
German-Brazilian Newspapers (GBN)^35^	65.12	65.76	64.99	70.72	70.58	79.49
S2-VL Dataset^10^	88.76	89.64	90.01	90.62	91.19	93.27
IIIT AR 13K^33^	89.04	89.12	89.62	90.37	91.41	92.41
Publaynet^32^	88.97	89.71	89.98	91.11	91.37	91.93

The most striking results are observed on datasets with highly complex and irregular layouts. On the PRImA^34^ Newspaper Dataset, D-REEL achieves an SSC score of 81.57, which is a remarkable improvement of more than 10 points over the next-best model, DocLayLLM^37^ (71.37). A similar substantial lead is seen on the German-Brazilian Newspapers (GBN)^35^ Dataset, where D-REEL scores 79.49 nearly 9 points higher than the strongest competitor. This vast performance gap on challenging documents strongly validates the efficacy of our embedding-based approach for modeling non-trivial spatial and semantic interdependencies where other methods falter.

This superior performance is consistently maintained across all other benchmarks. On the S2-VL, IIIT AR 13K, and Publaynet datasets, D-REEL scores 93.27, 92.41, and 91.93, respectively, consistently outperforming all other models. While the margin is narrower on these more structured document types, the consistent top-ranking performance underscores the robustness and versatility of our model.

*F1-score for the individual components of an article* The F1-scores for the individual components of an article including Title, Sections, Figure, Caption, Equation, and Table are presented in Table 3. D-REEL achieves superior F1-scores across all components when compared to existing state-of-the-art methods. The most significant improvement is observed for Figure and Caption components, where D-REEL demonstrates an improvement of approximately 6% over the second-best performing model, DocSAM. For the remaining components, a consistent improvement of approximately 2–3% is also observed.

**Table 3:** F1 score for the individual components of an Article.

Model / Components	Title	Sections	Figure	Caption	Equation	Table
LayoutLMv3^6^	85.78	94.12	89.72	94.38	89.87	90.64
YOLOv8^30^	86.05	95.96	88.46	95.71	93.06	93.77
LayoutLLM^36^	87.44	95.16	88.31	89.68	90.72	93.25
DocSAM^28^	88.06	96.87	88.91	90.15	93.73	94.14
D-REEL	93.62	97.27	94.39	97.49	96.26	96.41

### Qualitative results

**Fig. 2:**
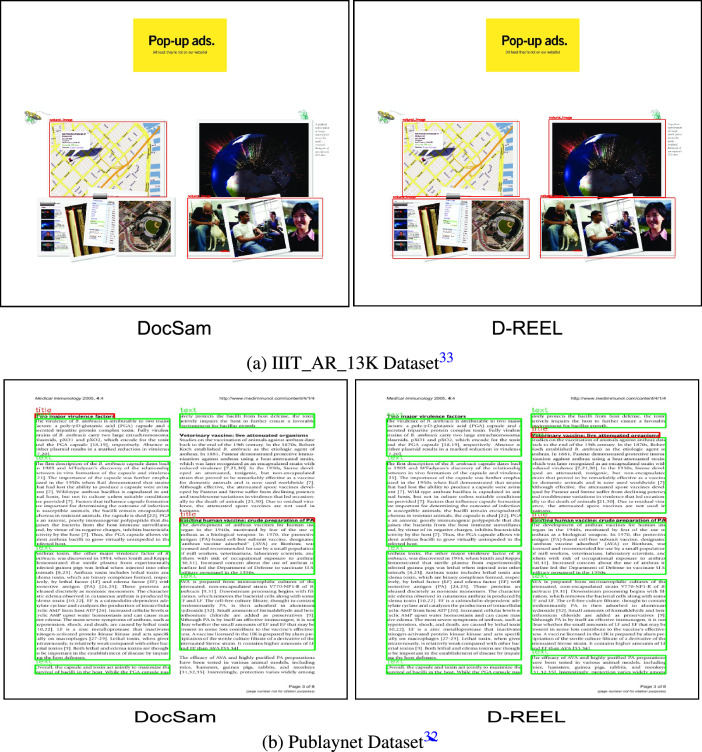
Result on Structured Dataset.

The visualization detection results of D-REEL with DocSam for two datasets (IIIT AR 13K, Publyanet) are shown in Fig. 2.

*Detection comparison* Figure 2a presents a qualitative comparison between DocSAM^28^ and our proposed D-REEL model on the IIIT_AR_13K^33^ dataset. While both models are able to identify and segment key document components, DocSAM^28^ exhibits misalignments and incomplete coverage of certain regions, particularly in complex layouts containing multiple overlapping visual and textual blocks. In contrast, D-REEL produces cleaner and more precise boundaries, capturing the structural hierarchy of document elements with greater accuracy. The improved localization is especially evident in regions containing image clusters and advertisement banners, where D-REEL preserves finer layout details that DocSAM^28^ tends to merge or partially miss. These qualitative results reinforce the quantitative improvements, confirming that D-REEL not only achieves higher evaluation metrics but also provides more reliable and visually consistent document segmentation outputs in practice.

In Fig. 2b, we present qualitative comparisons on the PubLayNet dataset, which contains complex scientific articles featuring structured layouts with titles, paragraphs, and section headers. DocSAM^28^ is able to detect most text blocks but exhibits inconsistencies, such as partially fragmented bounding boxes around multi-line paragraphs and occasional misclassification at the title level. In contrast, D-REEL delivers more coherent and tightly aligned segmentations. The bounding regions are consistently drawn around full paragraphs, with clearer separation between section headers and body text. This improved granularity ensures that logical document structures are better preserved, facilitating downstream tasks such as semantic segmentation and content extraction. The qualitative improvement on PubLayNet thus reinforces the advantage of D-REEL in handling detailed and information-dense layouts that are typical of scholarly and professional documents.

**Fig. 3:**
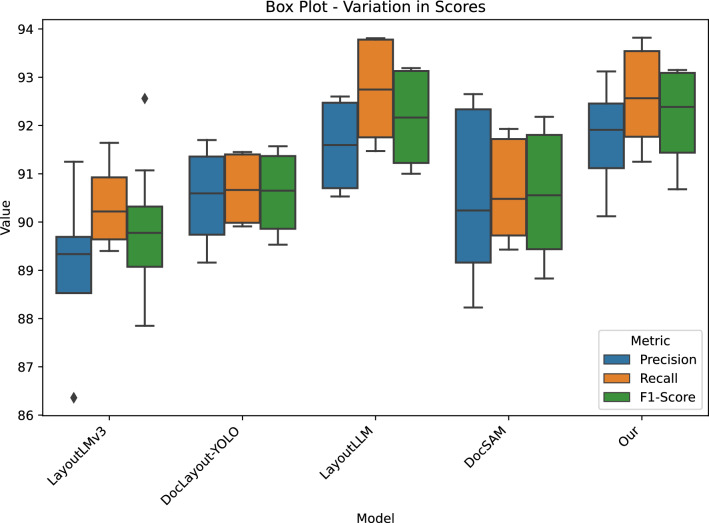
Box plot of Precision, Recall, and F1-Score across different document understanding models. The boxes represent the interquartile range (IQR), the horizontal bar shows the median score, and the whiskers extend to the minimum and maximum values. Outliers are depicted as individual points.

*Performance comparison* The box plot in Fig. 3 provides a comparative summary of the performance of five document understanding models LayoutLMv3, DocLayout-YOLO, LayoutLLM, DocSAM, and our proposed method (D-REEL) across the three evaluation metrics of Precision, Recall, and F1-Score. All models demonstrate relatively strong performance, with values generally concentrated between 89% and 94%, reflecting the maturity of current approaches in structured document understanding. However, the spread and central tendency reveal important differences in reliability and robustness among the models.

LayoutLMv3 and DocLayout-YOLO exhibit slightly lower medians compared to the other models, with narrower interquartile ranges (IQRs), suggesting relatively stable but modest performance. LayoutLMv3 also shows the presence of an outlier at the lower end, which highlights potential difficulties on certain datasets. LayoutLLM, on the other hand, achieves higher overall medians with compact distributions, suggesting not only improved performance but also consistent reliability across datasets.

In contrast, DocSAM presents greater variability, as shown by wider boxes and whiskers. While its median performance is competitive, the results reveal sensitivity to dataset differences, indicating fluctuations in precision and recall depending on document type. Finally, our proposed model, D-REEL, consistently outperforms the baselines. Its distributions are shifted upward for all metrics and show reduced variance, demonstrating both higher effectiveness and improved stability.

### Ablation study

To systematically analyze the contributions of layout detection, semantic relationship modeling, and field-level information extraction, we conducted an extensive ablation study across multiple datasets. For document layout and relationship modeling, we conducted experiments on multiple document datasets. The final model integrates three embeddings: image, OCR, and layout to generate comprehensive entity representations. To understand the contribution of each modality, we tested three two-modality variants: 1. Image + OCR embeddings – Combines visual features with text information. 2. Layout + OCR embeddings Combines spatial layout with text features. 3. Image + Layout embeddings Combines visual and spatial features without OCR. For other datasets focused on field-level information extraction, we evaluated detection performance independently to isolate its impact from higher-order semantic modeling.

#### Impact of D-REEL on invoice/receipt dataset

We have performed ablation study of D-REEL with other Invoice/Receipt datasets for document layout detection task only. The details of these datasets are described below.

*SROIE (scanned receipt OCR and information extraction)* Contains 626 receipt images with diverse layouts^38^. We detected key regions such as company name, address, date, and total fields across 234 unique templates, addressing challenges posed by template diversity and OCR noise.

*FUNSD (form understanding in noisy scanned documents)* Comprises 199 scanned forms with irregular structures and handwritten annotations^39^. We performed detection of key-value regions, leveraging layout cues to handle 81 unique templates with noisy and degraded scans.

*CORD (consolidated receipt dataset for post-OCR parsing)* Includes 1000 real-world receipts with highly varied layouts and multilingual text (primarily Korean with some English)^40^. D-REEL detection process identified structured regions (date, total price, item list) across 300+ unique layout templates, handling complex and visually degraded receipts.

*Quantitative result on invoice/receipt dataset* The Precision, Recall, and F1-Score results are detailed in Table 4. These performance metrics were evaluated on the FUNSD^39^, CORD^40^, and SROIE^38^ datasets. D-REEL showcases consistently strong and competitive results against other leading methods. It achieves a state-of-the-art F1-Score of 90.68, 91.69 and 93.09 on the FUNSD, CORD and SORIE dataset respectively. This performance validates D-REEL’s robustness and effectiveness for key document analysis tasks.

**Table 4:** Precision, Recall, and F1-Score across datasets for different document layout models.

Dataset	LayoutLMv3^6^			DocLayout-YOLO^30^			LayoutLLM^36^			DocSAM^28^			D-REEL		
	Precision	Recall	F1	Precision	Recall	F1	Precision	Recall	F1	Precision	Recall	F1	Precision	Recall	F1
FUNSD^39^	89.42	90.72	90.07	88.93	90.01	89.97	90.03	91.11	90.57	88.23	89.43	88.83	90.12	91.25	90.68
CORD^40^	88.36	89.40	87.85	89.16	88.91	88.53	90.76	91.85	91.30	89.47	89.82	89.64	91.45	91.94	91.69
SROIE^38^	89.25	89.72	88.48	91.26	91.40	91.33	91.60	93.78	92.68	92.65	91.72	92.18	92.37	93.82	93.09

**Fig. 4:**
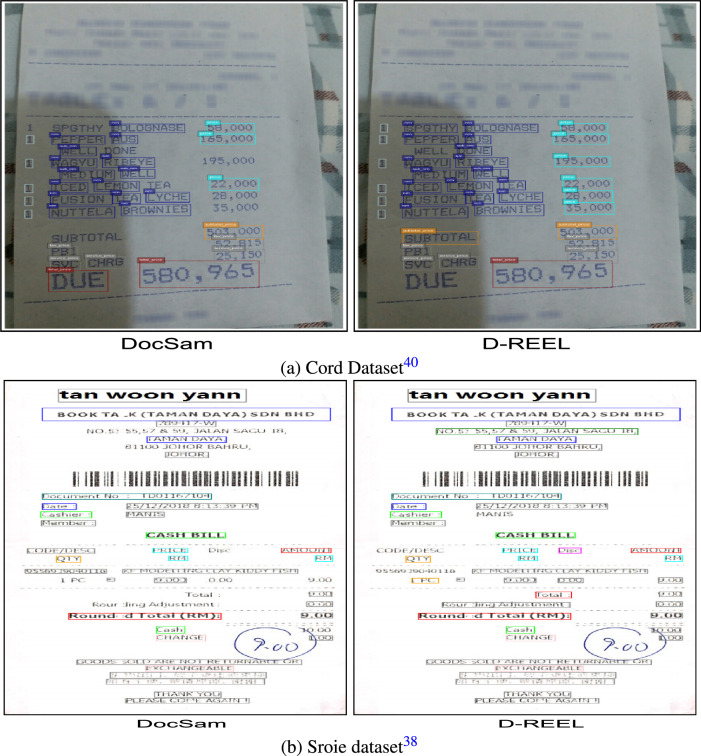
Result on unstructured dataset.

*Qualitative result on invoice/receipt dataset* In Fig. 4a, we illustrate qualitative results on the CORD dataset to compare DocSAM with our proposed D-REEL model. The receipt-style documents in this dataset pose challenges due to their small fonts, dense tabular structures, and close alignment of text with numerical entries. As observed, DocSAM often produces less accurate bounding regions, with several text items either partially detected or misaligned, especially within line-item entries and subtotal regions. On the other hand, D-REEL demonstrates more precise localization and segmentation, accurately capturing each individual text block while preserving the layout structure. The improvement is most evident in the recognition of tabular elements such as item descriptions and price values, where D-REEL maintains consistent bounding boxes that align tightly with textual content. This enhanced fidelity in detection and segmentation contributes directly to improved downstream information extraction, highlighting the effectiveness of D-REEL in structured financial documents such as receipts and invoices.

In Fig. 4b, we show qualitative results on the Sroie dataset, which primarily consists of scanned receipts containing structured key-value pairs such as merchant name, address, date, item details, and total amounts. While DocSAM is able to capture the majority of text instances, it often produces fragmented or loosely fitted bounding boxes, particularly in regions containing critical fields like totals, round off amounts, and key-value tags (e.g., “DATE”, “CASH”, and “TOTAL”). This can result in unnecessary overlaps and misalignments that affect downstream information extraction accuracy. In contrast, D-REEL demonstrates tighter and more consistent bounding boxes that correspond directly to semantic entities, reducing noise around field boundaries and better distinguishing between headers, itemized entries, and final totals. The enhanced regularization of structured document fields not only improves visual clarity but also directly benefits named entity recognition and key information extraction pipelines in end-to-end receipt understanding tasks. These improvements confirm the capability of D-REEL to generalize effectively to semi-structured financial documents such as receipts and invoices, outperforming baseline models in both accuracy and practical usability.

#### Impact of using pair wise modality

As per out default setting we have taken into consideration three modalities: OCR, Layout, and Image. To evaluate the impact of reduced information, we conducted experiments using only a pair of modalities (specifically, OCR and Layout) and measured the resulting performance of D-REEL on the SSC metric. Our observations indicate that the removal of a single modality consistently resulted in a performance reduction of up to 8% (particularly for PRImA dataset). This clearly highlights the critical importance of integrating all three modalities for optimal performance.

*OCR, layout only modality* The results for the OCR and Layout only configuration, measured using the SSC metric, are presented in Table 5. Even with this reduced modality setting, D-REEL demonstrates superior performance. It achieved the highest SSC score on four out of the five evaluated datasets, reaffirming its robust capabilities. The model’s advantage is particularly pronounced on the complex layouts found in the PRImA and GBN newspaper datasets. Although placing a close second on the IIIT AR 13K dataset, D-REEL’s consistently high scores across all benchmarks underscore its proficiency in effectively integrating explicit textual content with spatial information to accurately interpret a document’s underlying logical structure. However, a decrease in SSC score is noticed by excluding image modality.

**Table 5:** Semantic Structural Congruence (SSC) across datasets for different document layout models with input embeddings: OCR and Layout Only.

Dataset / Model	LayoutLMv3^6^	DocLayout-YOLO^30^	DocSAM^28^	LayoutLLM^36^	DocLayLLM^37^	D-REEL
PRImA Newspaper Dataset^34^	57.14	60.27	58.63	63.42	64.19	**73.05**
German-Brazilian Newspapers (GBN)^35^	58.08	60.72	59.37	62.31	63.57	**71.48**
S2-VL Dataset^10^	83.52	86.11	86.43	88.59	89.03	**90.47**
IIIT AR 13K^33^	84.68	87.29	87.15	89.21	**90.07**	89.14
Publaynet^32^	84.03	86.42	89.17	88.26	89.08	**89.31**

*Image, layout only modality* The results for the Image and Layout only configuration, measured using the SSC metric, are presented in Table 6. Even with this reduced modality. The results clearly establish the superiority of our D-REEL model, which achieved the highest SSC score across all five datasets. This demonstrates a more advanced understanding of the semantic and structural relationships between document elements. The performance gap is particularly significant on the PRImA and GBN newspaper datasets, where D-REEL surpasses the next-best models by a substantial margin. This confirms our architecture’s exceptional skill at inferring logical reading order from purely visual and spatial cues. However, a decrease in SSC score is noticed by excluding OCR modality.

**Table 6:** Semantic Structural Congruence (SSC) across datasets for different document layout models with input embeddings: Image and Layout Only.

Dataset / Model	LayoutLMv3^6^	DocLayout-YOLO^30^	DocSAM^28^	LayoutLLM^36^	DocLayLLM^37^	D-REEL
PRImA Newspaper Dataset^34^	59.34	61.27	60.18	66.41	67.58	75.42
German-Brazilian Newspapers (GBN)^35^	61.05	62.13	61.59	66.52	67.04	74.38
S2-VL Dataset^10^	85.53	87.42	88.16	89.03	89.57	91.49
IIIT AR 13K^33^	86.51	87.36	88.28	89.24	89.54	90.37
Publaynet^32^	87.11	88.44	88.53	89.57	90.19	90.58

*Impact of using Neighborhood Encoding (NE) modality* To further assess the contribution of the proposed Neighborhood Encoding (NE) module, we performed a targeted ablation comparing three variants of our framework: (a) the full D-REEL architecture with NE, (b) a version where NE is replaced by standard absolute 2D positional embeddings, and (c) a version with NE removed entirely. As shown in Tables 7 and 8 While baseline Transformer models such as LayoutLMv3 infer spatial structure solely through global attention and absolute coordinates, NE introduces an explicit relational inductive bias by learning nonlinear transformations of relative neighborhood geometry and fusing them with textual and OCR embeddings prior to the Transformer layers. This pre-attention relational encoding strengthens the model’s ability to interpret local layout structure, especially in visually degraded or irregular documents.

Across all datasets, and particularly on complex historical collections such as PRImA and GBN, removing or replacing NE results in clear performance drops SSC decreases by 6–11% and mAP by 1.5–3.8%. These results demonstrate that NE captures relational and topological cues that standard positional encodings fail to represent, enabling the model to remain robust even when OCR text is noisy or partially corrupted. The ablation confirms that NE plays a central role in stabilizing spatial reasoning and improving document understanding under real-world noise conditions.

**Table 7:** Ablation of the Neighborhood Encoding (NE) module on the Semantic Structural Congruence (SSC) metric.

Dataset / Model	Full D-REEL (NE) SSC	Std. 2D PosEnc SSC	No NE SSC
PRImA Newspaper Dataset	81.57	74.91	70.42
German-Brazilian Newspapers (GBN) Dataset	79.49	73.34	69.02
S2-VL Dataset	93.27	91.02	89.73
IIIT AR 13K	92.41	90.88	89.96
PubLayNet	91.93	89.71	88.15

**Table 8:** Ablation of the Neighborhood Encoding (NE) module on detection performance (mAP).

Dataset / Model	Full D-REEL (NE) mAP	Std. 2D PosEnc mAP	No NE mAP
PRImA Newspaper Dataset	90.64	88.91	87.12
German-Brazilian Newspapers (GBN) Dataset	90.09	88.37	86.25
S2-VL Dataset	95.12	94.23	93.88
IIIT AR 13K	93.28	92.34	91.85
PubLayNet	94.07	92.88	92.11

## Limitations and discussion

Although D-REEL achieves strong performance across multiple datasets, several limitations should be acknowledged. First, the explicit relationship supervision used for the SSC objective is based on only 200 manually annotated images. This limited annotation does not serve as the primary training signal, as SSC acts only as an auxiliary objective layered on top of large-scale multimodal pretraining from PubLayNet, S2-VL, IIIT-AR-13K, and DocBank. Moreover, the relational reasoning in D-REEL is driven largely by the Neighborhood Encoding (NE) module, which learns domain-invariant relative spatial patterns rather than memorizing instance-level annotations.

To further assess generalization beyond the annotated subset, we evaluated D-REEL on the DocGenome dataset, which contains millions of scientific pages and no relationship annotations. As shown in Table 9, D-REEL achieves the highest performance across all metrics (AP50 = 91.37, mAP = 90.89, SSC = 92.01), outperforming LayoutLMv3, DocLayout-YOLO, DocSAM, LayoutLLM, and DocLayLLM. This strong performance on a completely unannotated corpus demonstrates that SSC generalizes effectively and is driven by robust multimodal and spatial cues rather than the limited annotated relationship set. Nonetheless, future work could expand relationship annotations to additional domains such as legal, financial, or handwritten documents to further enhance coverage and evaluate potential domain-specific limitations.

**Table 9:** DocGenome performance comparison across detection (AP50, mAP) and Semantic Structural Congruence (SSC).

Model	AP50	mAP	SSC
LayoutLMv3	90.64	89.57	89.16
DocLayout-YOLO	89.11	88.94	89.12
DocSAM	89.67	88.16	89.12
LayoutLLM	–	–	90.62
DocLayLLM	–	–	91.48
Our (D-REEL)	91.37	90.89	92.01

**Fig. 5:**
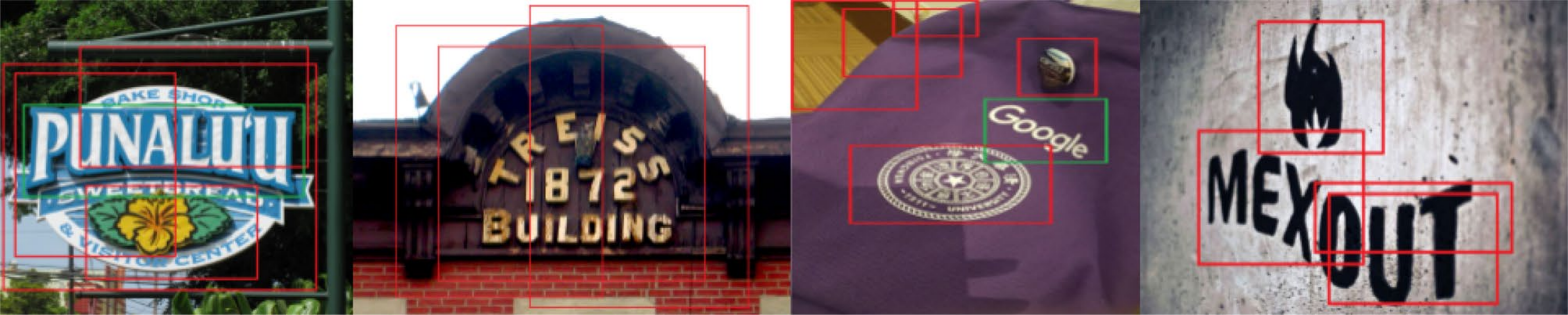
Failure case due to non-rectilinear layout: D-REEL struggles with artistic or radial diagrams that violate the rectilinear assumptions learned during training.

D-REEL significantly outperforms baselines on historical datasets like PRIMA and GBN, which contain degraded scans and substantial OCR noise. Although the Embedding Block employs a standard BERT encoder typically sensitive to OCR errors the robustness of D-REEL arises from four architectural mechanisms:

1. *Visual compensation* When OCR text is unreliable, the Vision Language Transformer redistributes attention toward visual cues (font size, weight, indentation, texture) and spatial signals from bounding boxes. This allows the model to recognize visually salient structures (e.g., titles) even when the textual signal is corrupted.
2. *Structural context (Neighborhood Encoding)* The Neighborhood Encoding block utilizes self-attention to model relationships between entities. Even if a specific node contains noisy text, its identity is often resolved by its context. The self-attention mechanism propagates this structural information, effectively “smoothing” out local noise.
3. *Spatial invariance* Bounding box embeddings originate from detection geometry and are unaffected by character-level OCR degradation. These stable spatial signals anchor the model’s predictions despite noisy text.
4. *Rectilinear layout bias* As shown in^41^ Fig. 5 D-REEL’s failure on non-rectilinear or stylized document layouts can be theoretically attributed to the geometric inductive biases embedded in both its spatial encodings and the Neighborhood Encoding (NE) module. The coordinate, area, and relative-position embeddings assume an underlying Euclidean grid in which semantic relationships correlate with axis-aligned proximity, and the NE module reinforces this assumption by computing attention weights over pairwise similarities derived from these rectilinear features. As a result, the model effectively learns a rectilinear manifold of document structure. When confronted with radial, curved, or otherwise non-linear layouts, this representational assumption breaks down: spatial coordinates produce non-isometric distortions, relative distances lose semantic meaning, and NE attention weights become unstable because the spatial cues contradict the learned rectilinear priors. This mismatch between input geometry and representational assumptions leads to degraded relational inference, highlighting the need for more geometry-aware or layout-invariant positional encodings in future work.

## Conclusion

In this paper, we introduced a novel representation learning approach for semantic correlation in structured documents. D-REEL method addresses the challenge of extracting and correlating information across documents with varied layouts, focusing particularly on the inter field relationships that are crucial for understanding documents such as invoices, research papers, and purchase orders. By leveraging position embeddings and incorporating a location agnostic method for scoring semantic correlation, we have significantly improved the accuracy of correlation scoring and extraction performance across different domains. D-REEL approach utilizes a neural network based model that generates dense embeddings for extraction candidates and their corresponding fields. The spatial relationships within documents are preserved and used to learn meaningful associations between fields, which allows for more robust and interpretable extraction processes. Additionally, the introduction of the Semantic Structural Congruence (SSC) metric has demonstrated substantial improvements in correlation accuracy over traditional methods. Experiments on public datasets, including those with complex layouts, show that D-REEL outperforms existing baseline techniques, offering improved F1 scores and providing valuable insights into the structure of documents. Furthermore, D-REEL allows for easy adaptation to diverse layouts and domains, making it a highly versatile solution for information extraction tasks. Future work could explore further refinements to the model, particularly in the context of handling more diverse document types and enhancing the interpretability of the learned embeddings.

## Data Availability

his study uses publicly available dataset which can be downloaded from 1. PRImA Newspaper: https://www.primaresearch.org/datasets/ENP, 2. German-Brazilian Newspapers: https://web.inf.ufpr.br/vri/databases/gbn/, 3. S2-VL: https://github.com/allenai/VILA, 4. IIIT AR 13K: https://cvit.iiit.ac.in/usodi/iiitar13k.php, 5. Publaynet: https://github.com/ibm-aur-nlp/PubLayNet (https:// www.cancerimagingarchive.net/collection/remind/) 6. Total-Text: https://www.kaggle.com/datasets/ipythonx/totaltextstr?resource=download all the datasets are cited in the manuscript. No new datasets were generated or analyzed during the current study. All data supporting the findings are included in the manuscript.
